# HTLV-1, the Other Pathogenic Yet Neglected Human Retrovirus: From Transmission to Therapeutic Treatment

**DOI:** 10.3390/v10010001

**Published:** 2017-12-21

**Authors:** Nicolas Futsch, Renaud Mahieux, Hélène Dutartre

**Affiliations:** 1 International Center for Research in Infectiology, Retroviral Oncogenesis Laboratory, INSERM U1111—Université Claude Bernard Lyon 1, CNRS, UMR5308, Ecole Normale Supérieure de Lyon, Université Lyon, F-69007 Lyon, France; nicolas.futsch@ens-lyon.fr (N.F.); renaud.mahieux@ens-lyon.fr (R.M.); 2 Equipe labellisée “Ligue Nationale Contre le Cancer”, France

**Keywords:** HTLV-1, HIV-1, viral transmission, ATLL, TSP/HAM, treatments

## Abstract

Going back to their discovery in the early 1980s, both the Human T-cell Leukemia virus type-1 (HTLV-1) and the Human Immunodeficiency Virus type-1 (HIV-1) greatly fascinated the virology scene, not only because they were the first human retroviruses discovered, but also because they were associated with fatal diseases in the human population. In almost four decades of scientific research, both viruses have had different fates, HTLV-1 being often upstaged by HIV-1. However, although being very close in terms of genome organization, cellular tropism, and viral replication, HIV-1 and HTLV-1 are not completely commutable in terms of treatment, especially because of the opposite fate of the cells they infect: death versus immortalization, respectively. Nowadays, the antiretroviral therapies developed to treat HIV-1 infected individuals and to limit HIV-1 spread among the human population have a poor or no effect on HTLV-1 infected individuals, and thus, do not prevent the development of HTLV-1-associated diseases, which still lack highly efficient treatments. The present review mainly focuses on the course of HTLV-1 infection, from the initial infection of the host to diseases development and associated treatments, but also investigates HIV-1/HTLV-1 co-infection events and their impact on diseases development.

## 1. Introduction

**Figure 1:**
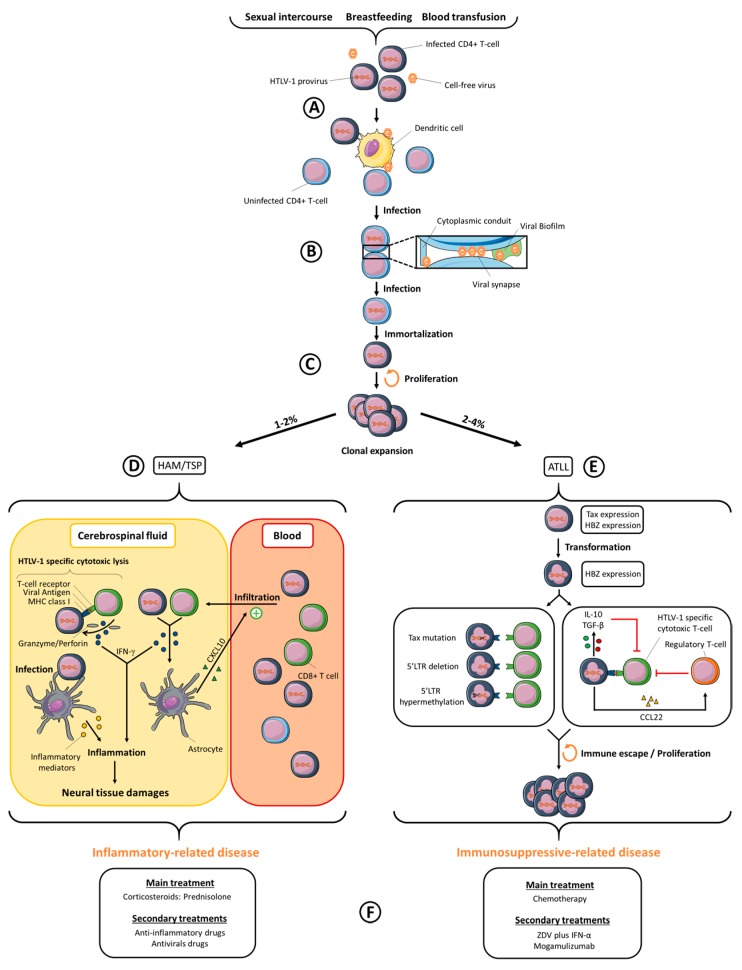
The course of HTLV-1 (Human T-cell Leukemia Virus type-1) infection. (**A**) HTLV-1 transmission through sexual intercourse, breastfeeding or contaminated blood products will allow the dissemination of HTLV-1 infected cells at the vicinity of dendritic cells, which are then able to transmit HTLV-1 viruses to CD4+ T-cells; (**B**) Neo-infection of CD4+ T-cells, which requires a cell-cell contact between an infected and a non-infected T-cell, is achieved through cellular conduits, the viral synapse or the viral biofilm; (**C**) Tax expression in HTLV-1 infected cells will result in consequent cell signaling alterations, such as continuous proliferation and apoptosis inhibition. Immortalized HTLV-1 infected cells will then proliferate through mitotic divisions, also known as clonal expansion; (**D**) TSP/HAM (Tropical Spastic Paraparesis/HTLV-1 Associated Myelopathy) is an inflammatory-related disease, which appears in 1 to 2% of infected individuals. The production of IFN-γ (Interferon- gamma), by HTLV-1 infected cells and HTLV-1 specific cytotoxic cells infiltrated in the cerebrospinal fluid, will lead to neural tissue damages, both directly by exacerbating inflammatory responses and indirectly by recruiting more IFN-γ producing cells in the CSF (Cerebrospinal fluid) through CXCL10 (C-X-C motif chemokine ligand 10) expression by astrocytes. (**E**) ATLL (Adult T-cell Leukemia/Lymphoma) is an immunosuppressed-related disease, which appears in 2 to 4% of infected individuals. The accumulation of genetic alterations in HTLV-1 infected T-cells leads to their transformation, and subsequent loss of Tax expression, either by mutations in the *Tax* gene or genetic modifications of the proviral 5′ LTR (Long Terminal Repeat). HTLV-1 transformed cells can escape from CTL (Cytotoxic T-cell) responses, through the loss of viral antigens presentation to the T-cell receptor, the production of Th1 (T-helper 1) inhibitory cytokines, and the recruitment of regulatory T-cells via CCL22 (C-C motif chemokine ligand 22) expression; (**F**) Current options to treat patients with TSP/HAM or ATLL.

Human T-cell Leukemia Virus type-1 (HTLV-1) was first described by Robert Gallo’s team in 1980 [1,2], before the discovery of Human Immunodeficiency Virus type-1 (HIV-1) [3]. HTLV-1 and HIV-1 are both retroviruses that emerged in human populations after zoonotic transmission from simian populations [4,5]. The number of HTLV-1-infected people was first estimated between 10–20 million people [6], and more recently between 5–10 million, even if the authors state that this number is likely underestimated [7]. HTLV-1 is not evenly distributed around the world but is found in highly endemic areas, such as Japan, sub-Saharan Africa, the Caribbean region and South America. Smaller infection foci are located in the Middle East, in Romania and in Australo-Melanesia [7]. In comparison, 37 million people are infected by HIV-1 worldwide, with the highest prevalence sites found in central- and South Africa [8], followed by the Caribbean region, Latin America, South-East Asia and Eastern Europe [9]. HTLV-1 and HIV-1 both lead to chronic infection. HTLV-1 infection may lead to the development of two main diseases: a malignant lymphoproliferation named Adult T-cell Leukemia/Lymphoma (ATLL) and a chronic progressive myelopathy named Tropical Spastic Paraparesis/HTLV-1 Associated Myelopathy (TSP/HAM). Other inflammatory syndromes linked to HTLV-1 infection were also reported. Approximately 2 to 4% of HTLV-1 infected individuals will develop an ATLL and 1 to 2% a TSP/HAM. HIV-1 is the etiological agent of an Acquired Immunodeficiency Syndrome (AIDS) that appears in most chronically infected people, independently of their age at the onset of infection, and which is fatal in the absence of treatment. Antiretroviral therapies (ART) have undergone great improvements during the last decades, notably by reducing or suppressing HIV-1 replication (and thus HIV-1 viral load) and by lowering the risk of developing AIDS [8]. Unfortunately, HTLV-1-infected people do not benefit from such efficient therapies yet: only symptomatic individuals are treated, with inconsistent benefits. This review will focus on the mechanisms of HTLV-1 viral transmission, HTLV-1-linked symptoms and their current treatments, drug development against HTLV-1 (summarized in Figure 1), but also on HIV-1/HTLV-1 co-infection.

## 2. Inter-Individual Viral Transmission

HTLV-1 inter-individual transmission has been associated with three main modalities: a vertical transmission from mother-to-child that occurs after prolonged breast-feeding [10], non-protected sexual intercourse [11] and contamination with blood products [12]. However, with systematic HTLV-1 antibody screening during blood collection and transfusion, initially established in Japan in 1986 and then in the United States of America, France, the Netherlands, Sweden, Portugal, Denmark, Greece, Ireland, Romania and the United Kingdom, the number of new HTLV-1 infections has significantly decreased. Even if these systematic controls are efficient in preventing HTLV-1 blood transmission, the cost-effectiveness of this strategy remains a debate in countries with a low prevalence [13]. From that perspective, other preventive options have been implemented. Because free viral particles are hardly found in the plasma of HTLV-1 infected individuals [14], the viral agent leading to inter-individual spread is presumably infected cells themselves. The nature of these cells is not fully confirmed, but they are likely to be CD4+ T-cells or macrophages [15]. It is estimated that HTLV-1 transmission linked to blood transfusion requires at least 90,000 HTLV-1-infected cells to promote infection in the recipient [16]. Thus, leukocyte reduction under this threshold would limit HTLV-1 incidence via blood transfusion. To limit HTLV-1 infection by prolonged breast-feeding or sexual intercourse, prevention policies and health advertisings have emerged. For example, HTLV-1 antibody screening of pregnant women, and further refraining breastfeeding by seropositive women, induced an efficient reduction in HTLV-1 mother-to-child transmission in Japan [17]. HIV-1 shares common entry routes with HTLV-1: HIV-1 is transmitted by parenteral exposure (blood transfusion, drugs injection), during sexual intercourse and by vertical transmission. However, in contrast to HTLV-1, HIV-1 transmission from mother-to-child also occurs during pregnancy, labor and delivery, in addition to breastfeeding. Several preventive strategies are available to limit HIV-1 transmission. Condom use, medical circumcision, pre-exposure ART and post-exposure ART (until 72 h after sexual intercourse) have greatly limited the risks of HIV-1 sexual transmission. ART of HIV-1 seropositive women and use of clean injection equipment have also effectively reduced newborn infection and parenteral transmission, respectively [8].

## 3. Cellular Transmission of HTLV-1

In infected individuals, HTLV-1 proviral DNA, i.e., HTLV-1 full-length integrated viral genome, is mainly found in activated CD4+ T-cells [18]. In addition, proviral DNA is also detected, but to a lesser extent, in other immune cell types, including CD8+ T-cells, B cells, monocytes, or dendritic cells [19,20,21]. HIV-1 shares a common in vivo tropism with HTLV-1, since CD4+ T-cells are major targets of HIV-1 infection. In vivo, latent HIV-1 proviruses are found in memory CD4+ T-cells, monocytes and macrophages, thus constituting viral reservoirs [22,23]. In vivo HIV-1 infection of dendritic cells has only rarely been reported [24,25] and blood dendritic cells do not seem to be targeted. Both viruses are nevertheless able to interact with dendritic cells in vitro, and this interaction, which may occur before T-cell infection in vivo, has been suggested as an important step for the subsequent infection of T-cells and further viral spread [26] (Figure 1A).

HTLV-1 and HIV-1 viral particles have been morphologically described [27]. Contrary to HIV-1 mature virions that harbor cone-shaped capsids, HTLV-1 mature virions show poorly defined polyhedral capsids, comprising both angular polygon-like and single-curved regions [27]. Differences are also observed in the trafficking of the structural Gag protein from both viruses, since HTLV-1 Gag is targeted to the plasma membrane at low concentration, whereas HIV-1 Gag first concentrates in the cytoplasm before being directed to the plasma membrane [28]. Although HTLV-1 infected cells are able to produce viruses when cultured in vitro, very few viral particles are found in vivo, as previously mentioned, and HTLV-1 cell-free virions are poorly infectious, except towards dendritic cells [26,29,30], probably because these cells are more susceptible than T-cells to HTLV-1 infection [29]. HTLV-1 viral particles infectivity has a very short half-life of 0.6 h at 37 °C, partly due to the sensitivity of HTLV-1 envelope proteins to this reduction [31,32]. In addition, recombinant HTLV-1 viruses pseudotyped with VSV-G envelope proteins are still less infectious than recombinant HIV-1 viruses pseudotyped with the same envelope [33]. This suggests that the low infectivity of HTLV-1 cell-free viral particles is not only associated with the instability of its envelope protein, but also to the intrinsic properties of the core particle itself and/or to post-entry events. In fact, efficient HTLV-1 viral transmission requires tight contacts between an infected cell and a target cell [34]. Although HIV-1 cell-free virions are competent in the infection of target cells, cell-to-cell transmission is also more efficient than infection with cell-free virus and probably represents the main mechanism of HIV-1 spread in vivo [35].

In vitro, three cell-to-cell transmission mechanisms have been reported so far for HTLV-1: the viral synapse [36], the viral biofilm [37] and the cellular conduits [38] (Figure 1B). The viral synapse is defined as a virtual space in which viral particles are budding, resulting in their accumulation close to an uninfected cell’s plasma membrane [36]. The HTLV-1 Tax protein plays a significant role in the viral synapse formation, since Tax co-localizes at the microtubule-organizing center (MTOC) [39], and it up-regulates, among others, the expression of ICAM-1 (Inter Cellular Adhesion Molecule 1) [40,41], whose interaction with LFA-1 (Lymphocyte Function-associated antigen 1) on a non-infected cell favors cell-cell adhesion and reorientation of the MTOC at the site of cell-cell contact, where viral assembly is thus polarized [42]. Interestingly, HIV-1 infected cells also tend to enhance cell-cell adhesions, a process which increases viral dissemination between T-cells through viral synapses [43,44]. The viral biofilm consists of HTLV-1 viral particles retained at the infected T-cell surface by extracellular-matrix proteins, such as collagen and agrin, and other cellular proteins, such as tetherin and galectin-3. The removal of this structure from an infected cell almost abolishes viral transmission, thus suggesting that it plays a more efficient role than viral synapse for transmitting HTLV-1 [37]. Indeed, isolated biofilm purified from HTLV-1 infected cells is infectious, in contrast to HTLV-1 particles released in the supernatant of the same cells [29]. More than just a very efficient cell-to-cell transmission pathway, it is proposed that the viral biofilm could prevent recognition by antibodies by masking HTLV-1 epitopes. Viral biofilm structures were also observed in infected cells from patients after in vitro culture [37]. HIV-1 accumulation has also been observed near the surface of infected cells [45] in structures that were proposed as budding platforms, and that polarized toward the cell-cell contact [46], thus allowing viral transfer at the viral synapse [43,47]. One can thus hypothesize that HIV-1 accumulation could occur at the surface of infected cells in structures that could be defined as a viral biofilm, although this has not been published yet. Cellular conduits are an expansion of filopodium-like structures towards neighboring cells [48]. These conduits are also induced by the HTLV-1 regulatory p8 protein expression [38]. In addition, p8 induces LFA-1 clustering at the cell surface, which results in better cell-cell adhesion between infected and uninfected cells through ICAM-1/LFA-1 interactions, but also favors the number and the length of cellular conduits [38]. HTLV-1 viral particles were identified at the junction of conduits with uninfected cells. Cellular conduits could then act both in stabilizing cell-cell contacts and in developing poly-synapses. HIV-1 uses similar mechanisms, since cell-cell transmission relies on filopodes, nanotubes and polysynapses, in addition to the viral synapse [45,49]. It was suggested that those mechanisms are mainly induced by the HIV-1 regulatory Nef protein [50].

## 4. Viral Dissemination

In vivo, HTLV-1 spread occurs through two mechanisms: neo-infection (the mechanisms of which were described above) or clonal expansion of infected cells [51]. Clonal expansion is believed to follow neo-infection (Figure 1C) and consists of mitotic divisions of HTLV-1 infected T-cells, which are immortalized by the expression of HTLV-1 oncoprotein Tax [52]. Interestingly when chronic infection has been established, viral replication is repressed either by direct inactivation of the viral genome [51], by cytotoxic immune responses targeting infected-cells [53] or by the indirect action of type I IFN (Interferon) produced by stromal cells [54] and able to inhibit the expression of viral proteins [54,55]. Furthermore, infected cells express tetherin [56], an IFN-induced gene that retains viral particles at the surface of infected cells, thus reducing the amount of particles released in the supernatant. All these processes probably contribute to the absence of cell-free viral particles in body fluids. Clonal proliferation of HTLV-1 infected cells thus remains the dominant contributor of HTLV-1 spread in vivo, since treatment of TSP/HAM patients with zidovudine and lamivudine, two reverse transcriptase inhibitors, does not reduce HTLV-1 proviral load [57]. Interestingly, 1- or 2-LTR DNA circles, which reflect active HTLV-1 replication, are detected both in asymptomatic HTLV-1 carriers and in symptomatic patients, suggesting that viral replication is nonetheless maintained at low levels during the whole course of infection [58]. Several mechanisms could contribute to the lack of zidovudine effects on TSP/HAM patients proviral load [55,59,60,61]. First, neo-infection could only represent rare and isolated events. Second, the high number of viral particles transmitted through cell-cell contact could circumvent antiviral therapy efficiency, as observed for HIV-1 [62]. Finally, HTLV-1-infected cells can be found in organs that are poorly targeted by antivirals, such as lymph nodes [63], thymus [64], or bone marrow after the infection of bone–marrow hematopoietic stem cells [65], thus allowing persistent replication even under active retroviral treatment, as observed in HIV-1 infection [66].

In three recipients who acquired HTLV-1 infection following transplantation from an HTLV-1 infected donor, kinetics of early HTLV-1 infection suggested that neo-infection and clonal expansion [67] occurred in parallel [68]. However, it is worth noting that both therapeutic immune suppression inherent to the transplantation procedure, which might have favored neo-infection, and rapid administration of ART to transplantation-acquired HTLV-1 infected patients, which might have favored clonal expansion, have probably disturbed the balance between infectious spread and mitotic proliferation in these patients.

Dendritic cells are believed to play a significant role in the initial steps of HTLV-1 dissemination. Not only are dendritic cells more sensitive to HTLV-1 infection than autologous T-cells [29], they are also able to transmit HTLV-1 viruses to autologous CD4+ T-cells [30], possibly via DC-SIGN (Dendritic Cell-Specific Intercellular adhesion molecule-3-Grabbing Non-integrin) [69]. Even though mature dendritic cells are less susceptible to HTLV-1 infection and less prone to transfer HTLV-1 to T-cells than their immature counterpart [70], mature dendritic cells from infected patients are able to stimulate proliferation of autologous T-cells [71,72,73], thus probably sustaining clonal expansion. The significant role of dendritic cells in HTLV-1 spread has also been demonstrated in different animal models. First, the infection of mice depleted from dendritic cells with a recombinant HTLV-1 virus pseudo-typed with the murine leukemia virus (MLV) envelop showed lower HTLV-1 proviral load, compared to infection of mice without depletion of dendritic cells [73]. Secondly, infection of macaques with HTLV-1 viruses unable to infect dendritic cells, due to mutations in the regulatory p8 and p12 proteins, led to poor seroconversion rates [74].

Transfer of HTLV-1 virus from dendritic cells to CD4+ T-cells or between CD4+ T-cells themselves through neo-infection mechanisms generates several infected CD4+ T-cell clones, each clone being defined by a unique integration site of HTLV-1 provirus within the host genome. Tax-mediated immortalization of HTLV-1 infected T-cells, and their subsequent mitotic divisions, will lead to the presence of expanded clones of HTLV-1 infected T-cells in infected individuals, which may persist for decades [75]. Even though the mechanisms regulating HTLV-1 clonality in vivo, i.e., determining the diversity of infected cells with different HTLV-1 integration sites, are not fully understood, oligoclonal expansion rather than monoclonal expansion of a given infected cell, could predispose to HTLV-1-associated diseases [63]. Furthermore, several factors other than clonality index are potentially involved in HTLV-1-associated diseases development, including proviral load itself, the number of infected cells transmitted from one individual to another one, or the immune response. In addition, a retrospective study suggested that HTLV-1-associated diseases are not only sporadic events but also sometimes cluster in families [76], highlighting the role of environmental and genetic factors in disease development. Interestingly, individuals who acquired HTLV-1 through blood transfusion are more likely to develop TSP/HAM [77], while individuals who acquired the virus during breastfeeding are more likely to develop ATLL [78].

## 5. HTLV-1-Associated Diseases

### 5.1. ATLL

ATLL was described before HTLV-1 discovery [79], but the link between HTLV-1 infection and the disease was prompted only two years after HTLV-1 discovery [2]. ATLL is defined as a highly aggressive malignancy of HTLV-1 infected CD4+ T-cells that develops after long-term chronic infection (Figure 1E). It is classified into four clinical subtypes, described as smoldering, chronic, acute, and lymphoma subtypes [80]. This classification relies on a wide array of diagnostic criteria such as lymphadenopathy, splenomegaly, hepatomegaly, hypercalcemia, skin and pulmonary lesions or organs infiltration. Acute and lymphoma subtypes are more aggressive than smoldering and chronic subtypes, with a median survival time of approximately 9.5 months *versus* 43 months, respectively [81]. In addition, acute and lymphoma subtypes represent 60% and 20% of all ATLL subtypes, respectively [82]. Symptoms are numerous, and mostly depend upon ATLL subtype. Abdominal pain, diarrhea, ascites, jaundice, pleural effusion, cough, sputum, fever, unconsciousness states and/or opportunistic infections are common features associated with ATLL in patients. Leukemic cells possess an unusual morphology, with flower-like shaped nuclei [79]. Their phenotype is characterized by expression of CD2, CD3, CD4, CD5, CD25, CCR4 (C-C chemokine receptor type 4) but not of CD7 [83,84,85]. CADM1 (Cell Adhesion Molecule 1) is also highly expressed in ATLL cells, with a potential effect on cell-cell adhesion, tumor growth and organ infiltration [86]. These proteins constitute good biomarkers to evaluate ATLL progression, since CD7 down-regulation on HTLV-1-infected cells reflects disease progression during the course of the infection [87]. Another receptor, CCR7 (C-C chemokine receptor type 7), is a hallmark of ATLL aggressiveness, since it is only expressed on cells from aggressive subtypes [84].

Leukemogenesis results from several events that contribute to transformation of HTLV-1 infected T-cells. Mechanisms suspected to play a role in HTLV-1-induced leukemogenesis include differential mRNA expression, up-regulation and down-regulation of numerous micro-RNAs, cell-signaling alterations, somatic mutations, epigenetic deregulations or aneuploidy [88,89,90,91]. The Tax viral protein plays an important role in these processes. First, Tax is able to modulate viral and cellular gene expression by activating CREB (cAMP response element-binding protein)/ATF (Activating transcription factor), SRF (serum response factor) and NF-κB (nuclear factor-kappa B) dependent pathways [92,93]. Second, Tax prevents cell-cycle arrest and inhibits both DNA damage repair and apoptosis pathways. Thus, Tax favors both the proliferation of infected cells and the accumulation of genetic alterations. Finally, Tax has been shown to induce transformation of rodent fibroblasts [94,95], but also cell transformation in transgenic *Drosophila melanogaster* and mice expressing Tax [96,97]. Even though the expression of Tax alone seems sufficient for immortalization, Tax poorly transforms human T-cells [98]. As transformation of primary human lymphocytes by HTLV-1 is nevertheless possible [99], several HTLV-1 proteins probably synergize to induce T-cell transformation and further development of ATLL. Indeed, HBZ (HTLV-1 basic leucine zipper factor) transgenic mice develop T-cell lymphomas [100], thus demonstrating that HBZ also participates in the mechanism of HTLV-1 dependent cellular transformation. Numerous ATLL cells often present mutations in the *tax* gene [101] as well as hypermethylation or deletion of the provirus 5′ LTR (Long Terminal Repeat) [102,103], which normally drives transcription of HTLV-1 viral genes encoded by the positive strand, including *tax* (Figure 1E). In contrast, the HBZ-encoding gene, the transcription of which is driven by the 3′ LTR, is consistently expressed in all ATLL cases [104]. Interestingly, via its protein or its mRNA, HBZ promotes T-cell proliferation, suppresses Tax-mediated viral transcription though the 5′ LTR, inhibits NF-κB activity, apoptosis and autophagy, disrupts host genomic integrity though miRNA expression and impairs Th1 (T-helper 1) mediated antiviral immune response [105,106,107]. However, although it has numerous functions, HBZ does not support productive HTLV-1 replication, but rather appears to participate in HTLV-1-induced transformation and to facilitate HTLV-1 infected cells persistence. Thus, Tax and HBZ cooperate in the transformation of infected CD4+ T-cells. Indeed, HBZ induces T-cells to become Tregs, especially by up-regulating Foxp3 expression [108]. Interestingly, human CD4+ Foxp3+ cells can both be immortalized and transformed after expression of Tax [109], thus suggesting that Tax oncogenic properties in human cells may also require at least HBZ activity. In addition, immune responses are deregulated, especially through the induction of a tolerogenic state [110,111], either via the direct action of the anti-inflammatory cytokines IL-10 (Interleukin-10) [112] and TGF-β (Transforming growth factor-beta) [113] produced by infected cells themselves or via the indirect action of CCL22 (C-C motif chemokine ligand 22) produced by infected cells [114], which allows the recruitment of Tregs and the inhibition of HTLV-1-specific cytotoxic T-cell (CTL) responses [115] (Figure 1E). Furthermore, although Tax is highly immunogenic in infected individuals, especially through the Tax (11–19) epitope [116], Tax expression is lacking in most ATLL cells, allowing their escape from Tax-specific CTL responses in ATLL patients.

Regardless of the precise mechanism of human T-cells transformation, HTLV-1 remains one of the most human oncogenic viruses [117]. Mechanisms by which a virus causes cancer can widely differ. Even though HTLV-1 regulatory proteins Tax and HBZ have oncogenic properties, as described above, alterations of the infected cell micro-environment could also play a role in cancer development. Indeed, viral oncogenesis follows chronic infection, which indicates that oncoviruses are able to escape from host immune responses. Furthermore, a dampened chronic inflammatory response [118] could result in immunosuppression and thus favor cancer development.

### 5.2. TSP/HAM

In 1985, the association between HTLV-1 infection and TSP/HAM development was described in Martinique, a French department located in the Caribbean [119]. At that time, almost 60% of TSP patients were shown to be seropositive for several HTLV-1 antigens. Soon afterwards, another study highlighted the link between HTLV-1 infection and HAM in Japan [120]. It was further shown that TSP and HAM were the same disease, thus called TSP/HAM. TSP/HAM is a chronic inflammatory disease of the central nervous system. It is characterized by a progressive spastic weakness of the lower limbs, lower back pain, and bowel and bladder dysfunctions. Spinal cord lesions and myelin loss, characteristic of TSP/HAM, could be induced by direct viral cytopathic effects, by immune-mediated reactions or both. In vitro infection of astrocytes by HTLV-1 induces the secretion of pro-inflammatory cytokines that are thought to contribute to the occurrence of neuronal lesions. Other studies report an accumulation of HTLV-1-specific CTLs within cerebrospinal fluid (CSF) [121,122]. Inflammatory products released by those cells, such as IFN-γ (Interferon- gamma), may damage neighboring glial cells, neurons or astrocytes. The role of HTLV-1-specific CTLs in infected individuals is therefore quite ambiguous: on one side, these cells directly kill HTLV-1 infected cells but on the other side, their chronic release of inflammatory cytokines could have a detrimental side effect. It is also worth noting that an efficient HTLV-1-specific CTL response relies on HLA (Human Leukocyte Antigen) class I genotype specificity. Indeed, the presence of HLA-A*02 allele in the southern Japanese population is associated with a lower proviral load and a lower risk of TSP/HAM, whereas the HLA-B*54 allele is associated with a higher risk of TSP/HAM [123].

HTLV-1-infected CD4+ T-cells also accumulate in TSP/HAM patients CSF and are able to produce IFN-γ [124]. Interestingly, IFN-γ stimulates CXCL10 (C-X-C motif chemokine ligand 10) production by astrocytes, an inflammatory chemokine that will favor leukocyte homing to inflamed tissues. Thus, CXCL10 secretion by astrocytes may enhance the infiltration of HTLV-1 infected CD4+ T-cells and HTLV-1 specific CTLs in the CSF, triggering an inflammatory positive feedback loop [125] (Figure 1D). In this model, TSP/HAM results from detrimental interactions occurring between the central nervous system and the immune system. Interestingly, a specific IFN-inducible signature is observed in all blood immune cells from TSP/HAM patients and not in cells from infected yet asymptomatic individuals or from patients suffering from other inflammatory diseases such as systemic lupus erythematosus or multiple sclerosis. This reflects a complex deregulation of IFN production in vivo that could be more likely linked to disease development rather than protection from viral infection [126]. In addition, since this IFN-inducible signature is supported by the production of both type-II (i.e., IFN-γ) and type-I IFN, it also opens the debate regarding a beneficial antiviral or detrimental role of type-I IFN in HTLV-1 infection and symptoms development. 

### 5.3. Other Syndromes

Apart from ATLL and TSP/HAM, HTLV-1 infection can lead to several other diseases such as uveitis, conjunctivitis, sicca syndrome, interstitial keratitis, pulmonary diseases, infective dermatitis, arthritis, myositis, Sjögren’s syndrome, Hashimoto’s thyroiditis, Graves’ disease and polyneuropathies [127]. Often left aside in comparison to TSP/HAM and ATLL, these HTLV-1-associated diseases are quite numerous. An accumulation of HTLV-1 infected T-cells in the eyes, and the subsequent release of various inflammatory cytokines by these cells is considered to cause HTLV-1-associated uveitis for example [128]. The prevalence rate and the risk factors of these syndromes are poorly evaluated. Despite occurring frequently in TSP/HAM patients, these inflammatory syndromes can also appear in HTLV-1 carriers non-diagnosed for TSP/HAM or ATLL. HTLV-1-associated infective dermatitis, which is the main manifestation of HTLV-1 in children and diagnosed by exudative eczematous eruption, is frequently associated with further development of TSP/HAM and in some cases of ATLL [129,130]. However, because it is characterized by an exacerbated Th1 immune response and a higher HTLV-1 proviral load than in asymptomatic HTLV-1 carriers, HTLV-1-associated infective dermatitis is rather believed to be a risk factor of TSP/HAM development [131]. Apart from inflammatory manifestations, HTLV-1 infection can also lead to opportunistic infections in patients with ATLL such as *Strongyloides stercoralis*, crusted scabies, tuberculosis or leprosy [132].

## 6. HIV-1 and HTLV-1 Co-Infections

As mentioned above, HIV-1 and HTLV-1 share common routes of transmission and cellular tropism. In regards to the simultaneous prevalence of these viruses in different parts of the world, HIV-1/HTLV-1 co-infection has frequently been reported. One of the first studies reporting a co-infection has shown that approximately 7% of individuals with AIDS or an AIDS-related disease are also seroprevalent for HTLV-1 [133]. However, the distinction between HTLV-1 and HTLV-2, a closely related virus from HTLV-1, was not possible at that time, thus supporting an overestimation of the real prevalence of HIV-1/HTLV-1 co-infections. During the second half of the 1980s, several HIV-1/HTLV-1 co-infection events were reported in Europe, America, and Africa, especially in hemophiliacs, intravenous drug users, homosexuals and sexual workers [134]. Nowadays, HTLV-1 and HIV-1 co-infection is mainly investigated in South America and Africa [135,136,137,138,139,140,141,142], with prevalence ranging from 0.5 to 10.9% depending on the studies. Differences in regional endemicity, ethnic origin of the population, risk behaviors and study designs could account for such variability. The consequences of HIV-1 and HTLV-1 co-infection may act at two levels: the cellular level and the host level.

In vitro co-infection of a given CD4+ T-cell by both HTLV-1 and HIV-1 has been reported [143]. In addition, co-infection of HTLV-1-infected cells by HIV-1 is also possible [144], suggesting the potential presence of both viruses in the same CD4+ T-cells in co-infected individuals. Of note, all virally infected cells are resistant to re-infection by a similar type of virus. As an example, resistance to HIV-1 superinfection was described [145]. Resistance to HTLV-1 superinfection has also been suggested following the observation that during natural infection, most HTLV-1-infected T-cells contain only a single integrated provirus [146]. It was previously suggested that HIV-1 replication could be enhanced through HTLV-1 Tax protein expression. Indeed, Tax enhances NF-κB activity [147], which in turn recognizes responsive elements on the HIV-1 promoter [148]. This observation was later supported by another study, in which Tax promoted HIV-1 transcription in latent CD4+ T-cells [149]. In contrast, a daily treatment with recombinant Tax protein added to the culture medium of in vitro HIV-1-infected PBMCs (Peripheral Blood Mononuclear Cells) showed opposite effects, with an inhibition of HIV-1 replication up to 14 days after infection [150]. Furthermore, HTLV-1-infected cells produce a wide range of chemokines, such as MIP-1 α (Macrophage Inflammatory Protein-1 alpha), MIP-1 β or RANTES (Regulated on Activation, Normal T-cell Expressed and Secreted) [151], which could act in an autocrine loop to suppress HIV-1 replication [152]. However, the level of chemokines secreted by co-infected cells is difficult to evaluate in vivo, and the effect of HTLV-1 co-infection on HIV-1 replication and vice versa deserves better investigation. In particular, sequential infections, i.e., whether the individuals were first infected by HIV-1 or HTLV-1, the length of mono-infection, and the extend of cellular defects induced by mono-infection before co-infection may lead to different outcomes in terms of HIV-1 or HTLV-1 replication. Taken together, these studies do not allow conclusions on a positive or a negative regulatory effect of HTLV-1 on HIV-1 in co-infected individuals.

The effect of HIV-1/HTLV-1 co-infection on the pathological conditions seems clearer and converges with a worsening of symptoms linked to HIV-1 or HTLV-1 infection [134,153]. Although the number of documented HIV-1/HTLV-1 co-infected cohorts remains low, it is believed that HTLV-1 worsens HIV-1 infection by accelerating progression to AIDS or increasing mortality [154]. Because co-infected patients usually have significantly higher CD4+ T-cell counts than HIV-1 mono-infected patients, their AIDS diagnosis may be impaired, leading to a significant reduction of their survival time [154]. In addition, in comparison to HIV-1 mono-infected individuals, most HIV-1/HTLV-1 co-infected individuals are more likely to suffer from myelopathy, thrombocytopenia, bronchitis, urinary tract infection or opportunistic infection, regardless of the age, ethnicity or CD4+ T-cells count [155,156]. Several reports support that HIV-1/HTLV-1 co-infection can influence the development of HTLV-1-associated diseases, with a worsening impact of HIV-1 on the development of ATLL [157] or of TSP/HAM [158,159,160]. This could be due to the higher production of IL-2 and IFN-γ observed in HIV-1/HTLV-1 co-infected individuals compared to HIV-1 or HTLV-1 mono-infected individuals [161], together with the up-regulated levels of RANTES in HIV-1/HTLV-1 co-infected individuals cells [162]. This cytokine profile may thus favor a faster onset of myelopathies and neurological disorders in co-infected individuals.

## 7. HTLV-1 Treatment and Drug Development

### 7.1. ATLL

Since 1978, several clinical trials performed by the Japan Clinical Oncology Group aimed at improving chemotherapeutic treatments used to treat ATLL patients [163]. The first generation of chemotherapy, consisting of CHOP (cyclophosphamide, doxorubicin, vincristine and prednisone) or CHOP-like treatments, resulted in relatively poor outcomes. Almost 20 years later, clinical trials of LSG15 (Lymphoma Study Group) based regimens have proven more efficient. LSG15-based regimens are eight-drug regimens consisting of at least VCAP (vincristine, cyclophosphamide, doxorubicin and prednisone), AMP (doxorubicin, ranimustine and prednisone) and VECP (vindesine, etoposide, carboplatin and prednisone) [164]. Compared to biweekly CHOP treatment, the LSG15-based treatment shows better results on the 3-year overall survival (24% vs. 13%) and on complete remission rates (40% vs. 25%) [165]. However, this was not confirmed in a larger longitudinal study that included 1600 Japanese ATLL patients (with lymphoma or acute ATLL) from 2000 to 2009 [81]. In this large cohort, half of the patients were treated bi-weekly or tri-weekly with CHOP, while 31% received an LSG15-based treatment. A relatively poor benefit of LSG15-based over CHOP treatment was observed. Regardless of the treatment, median survival times were 8.3, 10.6, 31.5, and 55 months for acute, lymphoma, chronic, and smoldering ATLL subtypes, respectively, while 4-year overall survival rates were 11%, 16%, 36%, and 52%, respectively [81]. This further highlights the poor efficiency of chemotherapy. In contrast, allogeneic hematopoietic stem cell transplantation following chemotherapy for patients with acute or lymphoma ATLL slightly improved the median survival time (14 vs. 6.7 months and 13.9 vs. 9.7 months, respectively) or the 4-year overall survival rates (27.8 vs. 6.8% and 32.3 vs. 13.7 months, respectively). Even though allogeneic hematopoietic stem cell transplantation constitutes an efficient treatment [166], ATLL patients resistant to chemotherapy are not able to receive this kind of transplantation. Furthermore, chronic and smoldering ATLL subtypes are considered indolent and are usually managed with watchful waiting. A long clinical follow-up of chronic and smoldering ATLL patients estimated a 15-year overall survival rate of 14.1%, with more than 60% of patients who died from an evolution of their indolent ATLL toward an acute ATLL. Thus, both new therapies development for aggressive forms of ATLL and better follow-up of indolent forms of ATLL are important.

To circumvent poor remission rates associated with chemotherapeutic treatments, efficacy of antiviral therapies has also been investigated. A treatment based on the combination of zidovudine (ZDV) and IFN-α showed high response rates in several clinical studies [167,168], especially when used as a first-line therapy in acute ATLL patients. A worldwide meta-analysis on the use of ZDV and IFN-α was then performed on about 250 ATLL patients [169]. This antiviral treatment increased the 5-year overall survival rate when used as a first-line antiviral therapy compared to chemotherapy (46% vs. 20%, respectively), but it decreased the survival rate when used after chemotherapy (12% vs. 20%, respectively). However, this effect was only observed in acute and chronic/smoldering subtypes, and not in the lymphoma subtype. Moreover, the 5-year overall survival rate reached 82% when complete remission of acute ATLL patients was achieved before antiviral therapy. Thus, a combination of ZDV and IFN-α should be considered as the gold standard first-line therapy in leukemic subtypes of ATLL, and its use is already established in France, the United Kingdom and Florida, but not in Japan, where the Japanese national health insurance system did not approve its use. So far, the optimal duration for ZDV/IFN-α treatment is yet unknown. A recent clinical case reported a sustained complete remission of a patient with chronic ATLL, still 6 years after the end of a 5-year treatment [170]. To increase the clinical cure of ATLL patients, immunotherapies based on monoclonal antibodies targeting specific markers of ATLL cells were tested. As such, targeting CD2 [171] or CD25 [172] showed poor to no effect when used in combination with chemotherapy. In contrast, the CCR4 chemokine receptor seems an interesting target. Indeed, it is overexpressed on HTLV-1 infected cells, and in approximately 90% of ATLL cases [85]. In addition, CCR4 positivity on ATLL cells has been associated with unfavorable prognosis [173]. Thus, a humanized anti-CCR4 monoclonal antibody (Mogamulizumab) has been generated. This defucosylated antibody induces a strong antibody-dependent cellular cytotoxicity, because of its high affinity binding to effector cells. Mogamulizumab monotherapy showed clinically meaningful antitumor activity, with an acceptable toxicity profile, in patients with aggressive ATLL, who relapsed after at least one chemotherapy regimen [174]. These encouraging results fostered the use of mogamulizumab in combination with LSG15-based chemotherapy to treat aggressive ATLL patients [175]. An increased complete remission rate was observed with a combined therapy of LSG15-based regimen with mogamulizumab, compared to LSG15-based alone (52% vs. 33%), but without any change in the overall survival rate [175]. Although LSG15-based treatment plus mogamulizumab was found to be associated with adverse drug reactions, notably infusion reaction and skin rash, most of those are manageable. Surprisingly, it has been recently observed that skin rash development was associated with a better prognosis [176]. Further clinical investigations are now required to evaluate mogamulizumab’s activity in a larger cohort of ATLL patients.

In the last 5 years, several isolated studies have focused on additional promising drugs that inhibit ATLL cell proliferation or induce cell death by several mechanisms. Among them are chemotherapeutic molecules such as lenalidomide [177] or bortezomib [178]; plant-derived steroids, alkaloids or carotenoids [179,180]; pro-apoptotic molecules such as Bcl-2 (B-cell Lymphoma 2) inhibitors [181,182], CDK9 (Cyclin-dependent Kinase 9) inhibitor [183] or arsenic in combination with ZDV and IFN-α [184]; histone deacetylase, such as valproate; inhibitors of iron uptake such as antibodies directed against the Transferrin Receptor 1 [185]; p53 expression activator such as synthetic retinoid ST1926 [186]; or an HTLV-1-targeted gene editing zinc-finger nuclease [187].

### 7.2. TSP/HAM

Contrary to ATLL, for which current therapies are mainly targeting HTLV-1-infected cells (Figure 1F), the management of TSP/HAM mainly consists in treating clinical symptoms. The anti-inflammatory corticosteroid prednisolone remains a typical treatment, due to the inflammation-based manifestations of TSP/HAM [120]. Corticosteroids currently represent the most effective anti-inflammatory therapy for most chronic inflammatory diseases, both by inhibiting inflammatory gene expression and activating anti-inflammatory gene expression. For patients with TSP/HAM, treatment effectiveness can be evaluated by assessing the motor disability through the Osame Motor Disability Score [77]. Until now, corticosteroids effectiveness in patients with TSP/HAM has largely been controversial among clinical studies, with none of them using a placebo-controlled trial, nor evaluating the long-term efficiency of the treatment. A recent retrospective study, however, analyzed prednisolone-treated TSP/HAM patients and untreated TSP/HAM patients for several years [188]. Continuous uptake of low-doses of prednisolone by TSP/HAM patients improved their Osame Motor Disability Score both in short-term (less than 3 years) and long-term (more than 3 years) durations of treatment, in comparison to untreated TSP/HAM patients. Because corticosteroid treatments are neither efficient nor tolerated by some patients, alternative treatments using molecules with anti-inflammatory properties, such as cyclosporine A [189], pentoxifylline [190], danazol [191], or antiviral cytokines, such as IFN-α [192,193] or IFN-β [194], have been developed among others [195]. Aside from the general motor disability, other TSP/HAM-associated symptoms, such as spasticity, bladder dysfunctions, urinary tract infections, constipation, neuropathic and mechanical pains or erectile troubles are subject to a wide array of drugs [123].

Because the majority of TSP/HAM treatments are designed to suppress clinical symptoms rather than eliminate HTLV-1-infected cells, research is now focusing on drugs that could modulate anti-HTLV-1 immune response and induce a decrease in HTLV-1 proviral load. However, as infected cells mainly spread through mitotic divisions, infected cells may not expose viral antigens for immune recognition. One option thus consists in using additional drugs that first induce viral gene expression. For instance, valproate, a histone deacetylase inhibitor, induces HTLV-1 proviral gene expression and can therefore expose virus-infected cells to immune response, notably to HTLV-1-specific cytotoxic lysis. Valproate long-term treatment is safe, but does not improve motor disability [196]. Analysis of valproate efficiency also led to conflicting results regarding its efficiency on proviral load decrease. One study reported an efficient 24-fold decrease in the proviral load after 3 months of treatment [197], while another one reported no proviral load decrease after 2 years of treatment, nor any difference in the CD8+ lysis efficiency [196]. Interestingly however, in asymptomatic baboons naturally infected with STLV-1, treatment with valproate in combination with ZDV induced a very efficient decrease in proviral load, probably as a consequence of the STLV-1-specific cytotoxic lysis associated with viral expression, together with a prevention of neo-infection by the antiviral effect of ZDV [59]. Therefore, using valproate in combination with antiviral drugs should be considered as a preventive treatment of asymptomatic carriers, to limit the proviral load rise that precedes disease development. Interestingly, multiple combinations of valproate with prednisolone and IFN-I improve the clinical outcome of TSP/HAM patients, and more importantly, efficiently reduce HTLV-1 proviral load [198]. Finally, building upon the success of the CCR4-targeting therapy in ATLL, anti-CCR4 antibodies have also been tested in TSP/HAM patients. Interestingly, mogamulizumab effectively reduces HTLV-1 proviral load, spontaneous proliferation of CCR4-positive CD4+ and CD8+ T-cells, as well as pro-inflammatory cytokines production [199]. Thus, combined therapies using valproate deserve better evaluation since they could offer better therapeutic options. Finally, because the treatments aiming at reducing proviral load and clinical outcomes are not fully efficient, and because multiple ranges of symptoms are associated with TSP/HAM, a physiotherapeutic approach seems useful to reduce pain, flaccidity, sedentariness, bowel and bladder troubles, and to improve patients’ overall quality of life [200].

### 7.3. Other Symptoms

Uveitis treatment is based on topical and systemic corticosteroids, in addition to lubricating drops which are used to prevent subsequent ocular complications [127]. Dermatological conditions associated with HTLV-1 are quite numerous and treatment depends on the diagnosis. Patients with ATLL often develop opportunistic infections, due to their immunocompromised state, and the associated treatment (antibacterial, fungicide, anti-helminthic) depends on the nature of the pathogen [201]. A decreased efficiency of *Strongyloides stercoralis* treatment has been reported in HTLV-1-infected patients compared to HTLV-1 seronegative individuals [202]. This was mainly associated to a decreased IgE (Immunoglobulin E) secretion in HTLV-1-infected patients. In addition, other cytokines such as IL-4, IL-5 and IL-13, which potentiate anti-helminthic responses, also decrease during the course of HTLV-1 infection, especially through IFN-γ production [203], thus making HTLV-1 infection an important risk factor for *Strongyloides stercoralis* dissemination. Unfortunately, *Strongyloides stercoralis* infection stimulates HTLV-1 clonal expansion in asymptomatic individuals [204]. Similar to *Strongyloides stercoralis* infection, HTLV-1-infected individuals with infective dermatitis have an increased abundance of HTLV-1 positive clones [205]. Thus, it seems appropriate to develop specific treatments for HTLV-1-infected individuals against *Strongyloides stercoralis* and infective dermatitis [203].

### 7.4. HIV-1/HTLV-1 Co-Infection

As discussed above, HIV-1/HTLV-1 co-infection can worsen the clinical outcome of HTLV-1 infection. Indeed, the lifetime risk of TSP/HAM is higher in HIV-1/HTLV-1 co-infected patients than in HTLV-1 mono-infected patients. Moreover, this lifetime risk is even higher in co-infected patients under ART, which seems responsible for neurological complications [142,158,206]. It has been shown that ART composed of zidovudine, lamivudine and abacavir (or didanosine), initially prescribed to treat HIV-1 infection in HIV-1/HTLV-1 co-infected patients, triggers an increase in HTLV-1 proviral load [207]. While HTLV-1 stimulates lymphocyte proliferation without cytopathic effects, HIV-1 induces a severe lymphocytic depletion with intensive cytopathic activity [208]. Because CD4+ T-cells are targeted by both HIV-1 and HTLV-1, HIV/1HTLV-1 co-infection may complicate HIV-1 management, especially by “artificially” elevating the CD4+ T-cells count in co-infected individuals compared to HIV-1 mono-infected individuals. A decrease in the CD4+ T-cell count has long been considered as an evidence of progression to AIDS, and also an indicator determining when to initiate ART in resource-limited areas. Thus, co-infection by HTLV-1 may mask HIV-1 induced immunosuppression, and therefore could complicate the timing of ART initiation, worsening AIDS progression and favoring subsequent opportunistic infections. In conclusion, HTLV-1 serological status should be checked in all HIV-1 patients from HTLV-1 endemic areas, in order to start ART without taking into account the CD4+ T-cells count. However, as ART seems to worsen HTLV-1 infection, combined therapies targeting HTLV-1 in addition to HIV-1 infection should be considered.

## 8. Vaccine

There is no vaccine to prevent or cure HIV-1 or HTLV-1 infections. The idea of developing an HTLV-1 vaccine already emerged in the 1980s, and it seemed at first easier than developing an HIV-1 vaccine [6]. The feasibility of an HTLV-1 vaccine has been supported first by the worldwide genetic stability among HTLV-1 strains, secondly by successful vaccination in animal models, and finally by the presence of potent HTLV-1-associated immune responses in infected individuals [6]. When expressed by a vaccinia vector, the HTLV-1 envelope gene induces a partial protection against HTLV-1 infection in rodents [209], probably through the generation of neutralizing antibodies directed toward the viral envelope, thus responsible of an efficient active immunotherapy [210]. In line with this observation, newborns from HTLV-1-infected mothers are protected from HTLV-1 infection as long as breastfeeding allows the transfer of antibodies from the mother, indicating that neutralizing antibodies are likely to prevent HTLV-1 infection [211]. Despite this interesting asset, both HTLV-1 cell-to-cell transmission and clonal expansion of infected cells without expression of viral proteins may contribute to limit HTLV-1 envelope exposure in vivo.

A therapeutic vaccine based on autologous dendritic cells pulsed with CD8+-specific Tax antigens induced sustained immune responses and showed promising effects on three ATLL patients either by stabilizing disease progression or inducing a partial remission [212]. However, targeting Tax to induce immune responses may not be the most appropriate method, since Tax expression is barely detectable in ATLL cells. In contrast, HBZ is constitutively expressed in vivo and acts as a protective CTL target antigen [213]. Thus, an HBZ-vaccine, based on a vaccinia platform, was tested in mouse models and was shown to elicit specific T-cell responses and eradication of HBZ-induced lymphoma [214]. Currently, an anti-HTLV-1 lentiviral vector-based vaccine, encoding a unique polypeptide derived from Tax, HBZ, p12 and p30 viral proteins, has proven to be safe and efficient to induce a cellular response in mice. In addition, monocytes from ATLL patients, which were further differentiated in dendritic cells and infected by the lentiviral vector in vitro, were able to stimulate autologous CD8+ T-cells, with consequent expression of IFN-γ, TNF-α (Tumor Necrosis Factor alpha), IL-2 and perforin in vitro. These encouraging results now need to be confirmed in clinical trials.

## 9. Conclusions

More than three decades of HIV-1 and HTLV-1 studies have greatly improved knowledge regarding their viral cycle as well as the patients’ management. Yet, complete remission of both viruses is not fully achieved, especially with the absence of a preventive or prophylactic vaccine. Furthermore, the lack of an efficient treatment for HTLV-1-associated diseases greatly impedes HTLV-1 cure. Development of new drugs is thus strongly needed. This could, in particular, help to treat HIV-1/HTLV-1 co-infected patients for whom therapeutic options focused on HIV-1 currently worsen HTLV-1-associated clinical outcome. In addition, large-scale clinical studies of HIV-1/HTLV-1 co-infected patients are still necessary to better understand the mutual viral interplay and its influence on diseases development. Recently, an HTLV-1 taskforce was launched by the Global Virus Network, in order to expand epidemiological studies, to increase research on HTLV-1 persistence mechanisms, replication and pathogenesis, to discover effective treatments, but also to develop a vaccine against HTLV-1 [215]. This initiative will certainly boost HTLV-1 research, as this virus has frequently been outshined by HIV-1 in the scientific scene.
